# Number of Metabolic Syndrome Components Is the Central Predictor of the Impact of Metabolic Syndrome on Outcome of Percutaneous Nephrolithotomy in Staghorn Nephrolithiasis

**DOI:** 10.1089/end.2019.0404

**Published:** 2019-11-08

**Authors:** Peng Xu, Jia Wang

**Affiliations:** Department of Urology, Institute of Urology, West-China Hospital, Sichuan University, Chengdu, China.

**Keywords:** metabolic syndrome, percutaneous nephrolithotomy, number of metabolic syndrome components, safety

## Abstract

***Introduction:*** How to quantify the impact of metabolic syndrome (MetS) on percutaneous nephrolithotomy (PCNL) is unclear. We aimed to evaluate the quantified effect of the number of MetS components on the outcome of PCNL.

***Materials and Methods:*** In this retrospective cohort study, consecutive 606 patients with idiopathic staghorn renal stones undergoing PCNL were included. The participants were divided into two groups: MetS(+) and MetS(−). The number of MetS components were calculated as 0 to 5. Primary outcomes were stone-free rate (SFR) and overall complication rate.

***Results:*** MetS, obesity, hypertension, increased triglycerides (TG), decreased high-density lipoprotein cholesterol (HDL), and diabetes mellitus were found in 24.1%, 38.1%, 70.0%, 29.9%, 34.5%, and 26.4% of the patients, respectively. SFR values were comparable between groups. MetS resulted in a higher rate of overall complication (*p* < 0.001, odds ratio [OR] = 2.4, 95% confidence interval [CI] 1.67–3.69), blood transfusion, urosepsis, larger hemoglobin deficiency, and length of hospital stay. Multivariable analysis confirmed that fasting plasma glucose (FPG) (*p* = 0.033, OR = 1.164, 95% CI 10.22–1.348) and number of MetS components (*p* = 0.001, OR = 1.496, 95% CI 1.184–1.890) were independent risk factors, whereas HDL (*p* = 0.014, OR = 0.428, 95% CI 0.217–0.837) played an independent protective role. Compared with 0, having 3, 4, and 5 MetS components was associated with stepwise increase in complication rate (19.5% *vs* 34.2%, 41.5%, 62.5%, *p* = 0.027, 0.006, <0.001; OR = 2.1, 2.9, 6.9). Subgroup analysis showed that MetS(+) patients without complications were associated with lower systolic blood pressure, TG, and FPG (*p* = 0.010, 0.031, 0.002, respectively).

***Conclusions:*** The number of MetS components is the central predictor in assessing both inner severity of MetS and outer risk for PCNL. The number of MetS components is recommended to be calculated on a scale of 0 to 5. Three, four, and five MetS components increase risk for PCNL in a stepwise manner regardless of the presence or absence of obesity. MetS components should be controlled preoperatively.

## Introduction

Metabolic syndrome (MetS) is the coexistence of several cardiovascular and endocrine risk abnormalities (International Diabetes Federation, 2006). Its diagnostic criteria include obesity (body mass index [BMI] >30 kg/m^2^) with at least two of the following: hypertension (HT; blood pressure [BP] >130/85 mm Hg), diabetes mellitus (DM; fasting plasma glucose [FPG] >5.6 mmol/L), increased triglycerides (TG; >1.7 mmol/L), and decreased high-density lipoprotein cholesterol (HDL; <1.03 mmol/L in men or <1.29 mmol/L in women), or treatment for any one of these disorders.^1^

Prevalence and etiology between MetS and urolithiasis are positively correlated.^2^ Nevertheless, few studies have focused on the impact of MetS on urologic surgeries. MetS is concerned with higher perioperative complications in coronary artery bypass surgery and arthroplasty.^3,4^ However, the impact of MetS on urologic surgeries remains controversial. MetS has been reported to not increase the risk of cystectomy and prostatectomy.^5^ However, other studies have supported that MetS increased infectious complications for prostate biopsy.^6^

According to the 2018 European Association of Urology guidelines, percutaneous nephrolithotomy (PCNL) is the standard therapy for renal stones >2 cm, especially for those in the lower calix and staghorn.^7^ The impact of MetS on the clinical outcome of PCNL is controversial. With comparable stone size of 7.8 *vs* 7.6 cm^2^, Akman and colleagues reported that MetS did not increase stone-free rate (SFR) or complications,^8^ whereas Ahmet found that MetS increased major complications of PCNL (21.9% *vs* 10.3%).^9^ Another study focusing on small stones with an average of size of 3 cm^2^ found higher complication rate and lower SFR in patients with MetS than in patients without MetS.^10^ It is unclear whether stone size affects the impact of MetS on PCNL.

Latest literature has provided further insights by quantifying MetS components. A large Japanese cohort study showed that stepwise increasing risk for stone formation in patients with 0, 1, 2, 3, and 4 MetS components was 57.5%, 61.7%, 65.2%, 69.3%, and 73.3%, respectively.^11^ Similarly, West and colleagues showed that prevalence of urolithiasis in patients with 0, 3, and 5 MetS components was 3%, 7.5%, and 9.8%, respectively.^12^ Recently, Johans and colleagues^13^ performed the first initial quantified study to assess risk of MetS for myocardial complication following PCNL by number of MetS components in a retrospective cohort of 39,868 participants. They found that 0, 1 to 2, and 3 to 4 MetS components corresponded with a myocardial infarction rate of 0.6%, 1.0%, and 1.8%, respectively (*p* < 0.001).^13^ However, how the number of MetS components impacts general SFR and complication rate of PCNL is still unclear. Therefore, in this retrospective cohort study, we aimed to highlight the role of the number of MetS components on PCNL outcomes by testing the most size-varying type of staghorn stones.

### Patients and methods

Our study was approved by the Ethics Board of our department. Data were collected from January 2012 to October 2018. Totally, 3125 consecutive patients were prospectively registered and received 3192 sessions of PCNL in our urology department. Among them, 606 idiopathic renal staghorn patients without neurogenic bladder, horseshoe kidney, chronic urinary track obstruction, renal tubular acidosis, or other inducement disorders were recruited because MetS is important and research worthy in staghorn stones as MetS is prevalent in complex stones,^14^ and large staghorn stones seem to be more representative to test the impact of MetS on outcomes of PCNL. The patients were divided into two groups: MetS(+) and MetS(−). The diagnostic criteria of MetS followed the International Diabetes Federation 2006.^1^

The BMI, BP, FPG, TG, and HDL were recorded at the last preoperative moment and no earlier than hospitalization. The number of MetS components was calculated by the number of MetS components the patients were suffering from. Because of the numerous studies on HT and DM and the lack of evidence of dyslipidemia on nephrolithiasis, we differentiated TG and HDL as two independent components of MetS. Therefore, the MetS components were numbered as 0, 1, 2, 3, 4, and 5.

All patients underwent preoperative blood biochemistry analysis, urinalysis, urine culture, abdominal and pelvic noncontrast enhanced CT, and diuresis renography. PCNL was conducted under general anesthesia in prone position with the guidance of C-arm fluoroscopy by one surgeon. The skin-to-calix approach was obtained by gradual dilation of metal dilators to 24F. Lithotripsy of the staghorn stones was performed by one surgeon using fourth-generation EMS (Swiss LithoClast; EMS Electro Medical System, Nyon, Switzerland). Considering the comprehensiveness of staghorn stones, one 14F nephrostomy tube and 4.7F Double-J stent were inserted in every patient and were extracted on postoperative day 5 and week 2.

The primary outcomes were SFR and overall complication rate. Stone size was calculated by surface area (length × width × 3.14/4). Stone-free status was defined as the absence of residual fragments or residual fragments <4 mm, according to urinary plain film or CT performed 4 weeks postoperatively. Complications were recorded by Clavien–Dindo system. Postoperative fever was recorded if the patient's body temperature was higher than 38°C. Pain was recorded when pethidine was injected. Hemoglobin deficiency (g/L) was measured by blood routine analysis before and within 24 hours after PCNL to assess blood loss.

Data were analyzed by using SPSS 20.0 (IBM, Armonk, NY) software. Continuous variables were calculated as mean ± standard deviation and were analyzed by independent *t*-test. Categorical variables were analyzed by Chi-square test. Logistic regression was used for multivariable analysis. Statistical significance was confirmed as *p* ≤ 0.05.

## Results

MetS, obesity, HT, increased TG, decreased HDL, and DM in all patients were found at 24.1%, 38.1%, 70.0%, 29.9%, 34.5%, and 26.4%, respectively. Baseline age, gender, stone size, and preoperative urinary tract infection and initial SFR were comparable between groups (Table 1).

**Table 1.** The Effect of Metabolic Syndrome on Percutaneous Nephrolithotomy for Staghorn Nephrolithiasis

	*MetS(−),* N* = 460*	*MetS(+),* N* = 146*	p
Age (median, quartile) year	48 (40, 58)	50 (42, 58)	0.136
*n* (%)			
Male	49 (63.6)	77 (53.1)	0.113
Complete staghorn stone	179 (38.9)	63 (43.2)	0.362
Solitary kidney	55 (9.8)	16 (11.0)	0.681
Ipsilateral surgery history	121 (26.3)	34 (23.3)	0.467
Positive urinary culture	136 (29.6)	35 (24.0)	0.191
Two tracts	8 (1.7)	1 (0.1)	0.600
Overall stone-free status	232 (50.4)	85 (58.2)	0.101
SFR by CTs^*^	155 (51.5)	52 (59.8)	0.173
SFR by plain film	77 (52.7)	33 (55.0)	0.768
Overall complications	101 (22.0)	60 (41.1)	**<0.001**
Minor complications	101 (22.0)	60 (41.1)	**<0.001**
(I) Fever	45 (9.8)	26 (17.8)	**0.009**
(I) Pleura injury	11 (4.6)	4 (2.7)	0.813
(I) Pain	16 (3.5)	2 (1.4)	0.191
(I) Hyperglycemia	1 (0.2)	3 (2.1)	**0.045**^#^
(II) Blood transfusion	15 (3.3)	21 (14.4)	**<0.001**
(II) Urosepsis	14 (3.0)	11 (7.5)	**0.017**
Major complications	3 (0.6)	0 (0)	1^#^
(IIIa) Renal arterial embolism	1 (0.2)	0 (0)	1^#^
(IVb) Uroseptic shock	2 (0.4)	0 (0)	1^#^
Mean (SD)			
Stone size, cm^2^	13.4 (9.2)	12.4 (5.5)	0.220
Serum creatinine, μg/mL	91.4 (35.2)	91.6 (29.0)	0.952
Lithotripsy duration, minutes	48.0 (18.9)	50.2 (21.8)	0.232
Hemoglobin deficiency, g/L	7.6 (4.1)	8.1 (4.6)	**0.025**
Length of hospital stay, days	6.5 (1.9)	7.3 (3.0)	**<0.001**

Bold indicates values of statistical significance (p < 0.05).

^*^ Number of patients measured by post-PCNL CT were 314 MetS(−) and 87 MetS(+).

^#^ Were tested by Fisher exact probability test for the small number.

CI = confidence interval; MetS = metabolic syndrome; PCNL = percutaneous nephrolithotomy; SD = standard deviation; SFR = stone-free rate.

### SFR values were comparable between groups

Higher overall complication rate was found in the MetS(+) group compared with the MetS(−) group (*p* < 0.001, odds ratio [OR] = 2.4, 95% confidence interval [CI] 1.67–3.69) (Table 1). The increased complication rate was mainly caused by the higher rate of postoperative fever and blood transfusion. MetS also resulted in larger hemoglobin deficiency and longer hospital stay (Table 1).

Furthermore, individual roles of every component of MetS were analyzed in Table 2 to assess their different importance. Increased TG, decreased HDL, DM, obesity, and MetS resulted in higher risk of complications. Multivariable logistic regression found that FPG (*p* = 0.033, OR = 1.164, 95% CI 1.022–1.348) and the number of MetS components (*p* = 0.001, OR = 1.496, 95% CI 1.184–1.890) were independent risk factors, and HDL (*p* = 0.014, OR = 0.428, 95% CI 0.217–0.837) played an independent protective role.

**Table 2.** Analysis of the Relationship Between Overall Complications and Metabolic Syndrome Components

	*Overall complications*		*Univariable analysis*	*Multivariable analysis*		
HT	*+, (*N* = 161)*	*−, (*N* = 445)*	p	p	*OR*	*95% CI*
(+)	114 (26.9)	310 (73.1)	0.786	0.342	1.011	0.869–1.995
(−)	47 (25.8)	135 (74.2)				
TG						
(+)	70 (38.7)	111 (61.3)	**<0.001**	0.440	0.937	0.795–1.105
(−)	91 (21.4)	334 (78.6)				
HDL						
(+)	86 (41.1)	123 (58.9)	**<0.001**	**0.014**	**0.428**	**0.217–0.837**
(−)	75 (18.9)	322 (81.1)				
DM						
(+)	63 (39.4)	97 (60.6)	**<0.001**	**0.033**	**1.164**	**1.022–1.348**
(−)	98 (22.0)	348 (78.0)				
Obesity						
(+)	75 (32.5)	156 (67.5)	**0.01**	0.406	1.040	0.907–1.038
(−)	86 (22.9)	289 (77.1)				
MetS						
(+)	60 (41.1)	86 (58.9)	**<0.001**	**0.001**	**1.496**	**1.184–1.890**
(−)	101 (22.0)	359 (78.0)				

Bold indicates values of statistical significance (p < 0.05).

Data of complication were recorded by *n* (%). Univariable analysis was carried out by Chi-square test. Multivariable analysis was done by multivariable logistic regression on the absolute value of every patient to take more accurate information into consideration. In multivariable analysis, HT, obesity, and MetS were recorded of the systolic BP, body mass index, and number of MetS components.

BP = blood pressure; DM = diabetes mellitus; HDL = high-density lipoprotein; HT = hypertension; TG = triglycerides.

**FIG. 1.**
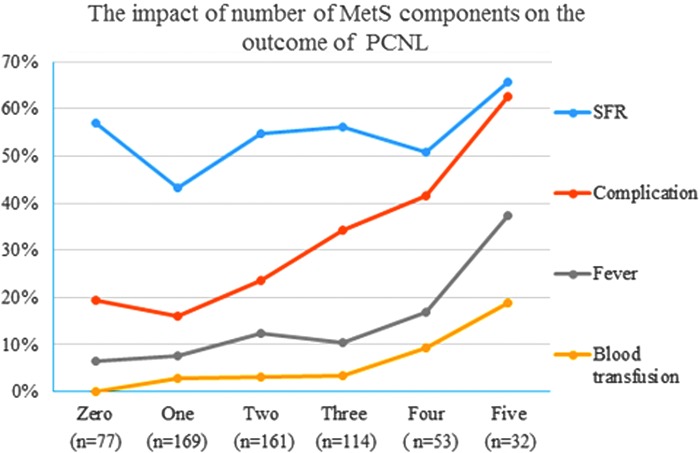
The impact of the number of MetS components on the outcome of PCNL. DM = diabetes mellitus; HDL = high-density lipoprotein; MetS = metabolic syndrome; PCNL = percutaneous nephrolithotomy; TG = triglycerides.

**FIG. 2.**
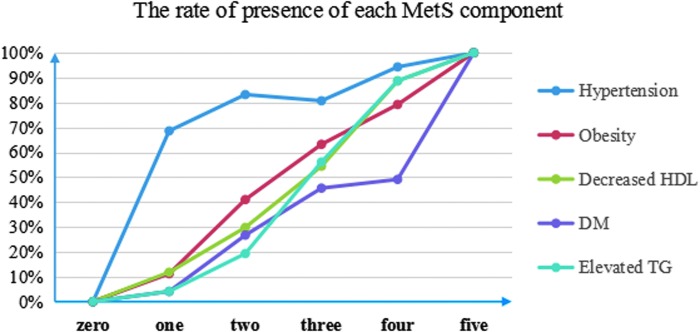
The rate of presence of each MetS component in different number of MetS components. SFR = stone-free rate.

Since the number of MetS components had the largest OR value, it was further analyzed (Fig. 1). The rate of overall complication, postoperative fever, and blood transfusion changed significantly as the number of MetS components increased from 0 to 5 (*p* < 0.001, *p* < 0.001, *p* < 0.001). Compared with 0, complication rates were comparable when the number of MetS components was 1 and 2 (19.5% *vs* 16.0% and 23.6%, *p* = 0.498 and 0.475) and increased significantly when the number of MetS components was 3, 4, and 5 (19.5% *vs* 34.2%, 41.5% and 62.5%, *p* = 0.027, 0.006, <0.001; OR = 2.1, 2.9, 6.9). No significant difference in SFR was found among patients with different numbers of MetS component (*p* = 0.085). The ratio of every MetS component increased as the number of MetS components increased (*p* < 0.001) (Fig. 2).

Subgroup analysis showed that compared with the MetS(−) group, MetS increased the complication rate in all groups with small, medium, and large stones (Table 3). For the presence or absence of obesity, patients with 3 and 4 MetS components were differentiated by whether they achieved the diagnostic criteria of MetS. Subgroup analysis showed that overall complication, blood transfusion, and postoperative fever rate in the MetS(+) group were comparable with the MetS(−) group (Table 4). The last subgroup was performed only within the MetS(+) group (Table 5). Compared with those without complications, patients with complications were found to have higher preoperative systolic BP, TG, FPG, and number of MetS components.

**Table 3.** Subgroup Analysis of Risk of Metabolic Syndrome on Percutaneous Nephrolithotomy in Different Size of Stones

*Subgroup*	*MetS*	*No. of patients*	*Complication (+, %)*	p	*OR*	*95% CI*
Large (>4 × 4 cm^2^)	(+)	64	29 (45.3)	0.028	1.939	1.071–3.509
	(−)	167	50 (29.9)			
Medium (3 × 3–4 × 4 cm^2^)	(+)	51	19 (37.3)	0.004	2.594	1.330–5.060
	(−)	204	38 (18.6)			
Small (<3 × 3 cm^2^)	(+)	31	12 (38.7)	0.004	3.692	1.454–9.376
	(−)	89	13 (14.6)			

CI = confidence interval; OR = odds ratio.

**Table 4.** Subgroup Analysis on the Impact of the Number of Metabolic Syndrome (MetS) Components Independent of the Diagnosis of MetS

	*MetS diagnosis*		p
	*(+)*	*(−)*	
Overall complication			
(+)	40 (65.6)	21 (34.4)	0.571
(−)	74 (69.8)	32 (30.2)	
Blood transfusion			
(+)	6 (66.7)	3 (33.3)	0.916
(−)	108 (68.4)	50 (31.6)	
Postoperative fever			
(+)	14 (66.7)	7 (33.3)	0.866
(−)	100 (68.5)	46 (31.5)	

Data were recorded by *n* (%). Patients of 3 and 4 MetS components were taken into analysis who were possible to reach the diagnostic criteria of MetS or not.

**Table 5.** Subgroup Analysis of the Relationship Between Metabolic Syndrome (MetS) Baseline Value and Overall Complication Rate in MetS Patients

	*Overall complications*		p
	*+ (*n* = 60)*	*− (*n* = 86)*	
Systolic BP	142.6 (15.0)	136.5 (12.8)	**0.010**
Diastolic BP	87.0 (9.4)	88.7 (7.4)	0.324
TG	2.6 (1.8)	2.0 (1.1)	**0.031**
HDL	1.0 (0.3)	1.1 (0.3)	0.070
FPG	6.1 (1.4)	5.4 (1.0)	**0.002**
BMI	32.4 (2.2)	32.4 (2.3)	0.921
Number of MetS components	3.97 (0.84)	3.56 (0.73)	**0.002**

Bold indicates values of statistical significance (p < 0.05).

Data were recorded by mean (SD).

BMI = body mass index; FPG = fasting plasma glucose.

## Discussion

A multidimensional relationship between urolithiasis and MetS was found in terms of prevalence, etiology, and impact on clinical outcomes. A spontaneous rising trend of urolithiasis and MetS has been reported. The prevalence of urolithiasis has grown from 3.8% in 1970s to 8.8% in 2000s in the United States and from 5.95% in 1990s to 10.63% in 2010s in China.^15,16^ Similarly, MetS increased from 27.9% to 34.1% in United States during 1996–2006^17^ and from 9.8% in 2009 to 14.4% in 2018 in China.^18^ Additionally, urolithiasis and MetS are mutual risk factors in etiology. Patients with a history of urinary stones are more likely to develop HT and DM (hazard ratio 1.29–1.69 and 1.32).^15^ Correspondingly, the presence of MetS, HT, and DM results in higher risk of developing urolithiasis (relative risk 1.78, 1.24–5.5, and 1.29–1.67, respectively)^15^ possibly because both urolithiasis and MetS share the same cause of insulin resistance, which results in hypercalciuria, hyperoxaluria, hyperphosphaturia, hyperuricosuria, hypocitraturia, and lower urine pH, thus facilitating calcium oxalate and urate stone formation.^19^ Our results revealed that MetS had a ratio of 24.1% in renal staghorn stones, which was more than 6.7% to 9.5% in general urinary stones.^9,13^ Our finding supported the association of MetS and severity of renal stones reported by Kohjimoto and colleagues^14^

With respect to clinical outcomes, Johans and colleagues performed the first study quantified by the number of MetS components, which showed that risk of post-PCNL myocardial infarction increased as the number of MetS components increased from 0 to 4 (Table 6).^13^ According to Table 1, our data showed that MetS affected PCNL mainly in safety instead of efficacy because of inflammatory and hemorrhagic disorders. These results were similar to those reported by Nalbant and colleagues.^10^ Additionally, the baseline of complete staghorn stones and positive urine culture, an independent risk of post-PCNL infection, was comparable between groups, supporting the specific effect of MetS on hemorrhage and urinary tract infections. Table 2 further confirmed DM and the number of MetS components as independent risk factors, whereas HDL was an independent protective factor.

**Table 6.** The Literature Review of the Impact of Metabolic Syndrome on Outcomes of Percutaneous Nephrolithotomy

*Research ID*	*Nation*	*Types of study*	*Participants*	*Outcomes*
Tefekli and colleagues^9^	Turkey	Retrospective cohort; October 2002–Feburary 2005	41 *vs* 389	Auxillary procedures ↑ (17.1% *vs* 11.3%, *p* = 0.048); Major complication ↑ (21.9% *vs* 10.3%, *p* = 0.03)
Akman and colleagues^8^	Turkey	Retrospective matched-cohort; 2002–December 2011	73 *vs* 73	Comparable SFR and complications; Recurrence of calcium oxalate monohydrate in 3 months postoperatively ↑ (43.3% *vs* 19.2%, *p* = 0.019); eGFR in 12 months postoperatively ↓ (87.8–66.6 *vs* 96.4–91.2 mL/min/1.73 m^2^)
Nalbant and colleagues^10^	Turkey	Retrospective cohort; May 2012–May 2014	80 *vs* 130	Auxillary ureteroscopy ↑ (5% *vs* 2.3%, *p* < 0.05); Residual stones ↑ (10% *vs* 2.3%, *p* < 0.05); Ureteral stent indwelling ↑ (22.5% *vs* 6.1%, *p* < 0.05); Fluoroscopy duration ↑ (9.8 ± 2.7 *vs* 4.9 ± 1.9, *p* < 0.05); Hct loss ↑ (4.1% ± 1.9% *vs* 2.6% ± 1.6%, *p* < 0.05); Hospitalization ↑ (3.5 ± 1.5 days *vs* 2.2 ± 1.1 days, *p* < 0.05); Urine leakage >24 hours ↑ (22.5% *vs* 6.1%, *p* < 0.05)
Johans and colleagues^13^	United States	Retrospective Cohort; 2007–2011	Patients with MetS comorbidities (0–1): (2–3): (3–4) = 17,932: 19,268: 2668	MetS comorbidities ↑ postoperative MI (0: 0.6%; 1 − 2: 1.0%; 3 − 4: 1.8%, *p* < 0.001); 3–4 MetS comorbidities ↑ postoperative MI (1 − 2: OR = 1.2, *p* = 0.147; 3 − 4: OR = 2.2, *p* < 0.001).

Participants and outcomes were presented by data of patients with MetS(+) *vs* the MetS(−).

eGFR = estimated glomerular filtration rate; MI = myocardial infarction.

First, HT produces hyaline degeneration in the arteriole, which thickens the arteriole wall and decreases blood irrigation. Second, the DM thickens the basement membrane in the capillary and is more likely to develop fast macrovascular atherosclerosis. Third, hypertriglyceridemia and hypercholesterolemia are known to induce atherosclerosis. HDL functions the efflux of cholesterol in macrophage-derived foam cells under angiothelium through ATP-binding cassette transporter G1 and ATP-binding cassette transporter A1 pathways, which has antiapoptotic, anti-inflammatory, and antioxidant effects against atherosclerosis.^20^ Therefore, patients with MetS are more prone to develop vascular lesions, which result in dysfunction of coagulation and higher blood loss during operations.

The other major risk of infectious complication caused by MetS may involve immune dysfunction by DM and dyslipidemia. Patients with obesity and DM are more likely to develop urinary tract infections (OR = 1.24, 95% CI 1.10–1.39)^21^ and are more likely to exhibit sever urinary infections, such as xanthogranulomatous pyelonephritis, emphysematous pyelonephritis, emphysematous cystitis, uroseptic shock, and death.^22^ High TG and low HDL decrease cellular immunity by reducing the function of dendritic cells and T lymphocytes.^23^ DM impairs humoral immune response by reducing total IgG and specific IgG.^24^ MetS causes a proinflammatory state and changes in extracellular matrix through higher C reactive protein, tumor necrosis α, and endothelin and plasminogen activator inhibitor-1, but lower nitric oxide by an excessive reactive oxygen species stress disorder.^25^

As shown in Figure 1, the complication rate significantly increased only when the number of MetS components was 3, 4, and 5 and were as high as 34.2%, 41.5%, and 62.5%, respectively. Our data suggest that differentiating dyslipidemia into increased TG and decreased HDL is important because 4 and 5 MetS components corresponded to complication rates of 41.5% and 62.5%, which is clinically important. Therefore, it is recommended that MetS components be calculated on the scale of 0 to 5. Our results were similar to the results of Johans and colleagues, which showed that 1 to 2 MetS components did not increase the risk of myocardial infarction, but the presence of 3 to 4 MetS components did (OR = 2.2, 95% CI 1.54–3.15).^13^ Therefore, the number of MetS components shows accumulative effect in risk for PCNL, implying that the number of MetS components should be identified as a predictor of risk for PCNL. As shown in Figure 2, the ratio of each component increased with the number of components, implying that the number of components can be considered as an intrinsic predictor for MetS severity. Therefore, the number of MetS components should be considered as the central marker while assessing both inner severity of MetS and outer risk for PCNL.

An interesting finding of the study is shown in Table 4. According to the definition of MetS, obesity is indispensable because it plays a decisive role. However, in the subgroup analysis of Table 4, safety outcomes of overall complication, blood transfusion, and fever were comparable between patients with 3 and 4 MetS components with and without obesity. This suggested that although obesity does play an important role in MetS, obesity does not play a decisive role in predicting the risk for PCNL. Our finding was in accordance with a latest systematic review published in 2017, which showed that superobesity increased operation duration but did not change the rate of either complication or stone-free status of PCNL.^26^

The results of the MetS(+) subgroup analysis showed that patients with complications were associated with higher systolic BP, TG, FPG, and number of MetS components than those without complications. This was similar to the findings of Zmistowski and colleagues who reported 49%, 8%, and 8% complication rates following total joint arthroplasty in patients with MetS, controlled MetS, and non-MetS.^27^ Thus, uncontrolled MetS should be regarded as a relative contraindication of PCNL. However, the recommended cutoff values require further studies.

Table 3 demonstrated that MetS increased risk for PCNL in all stone-size subgroups, which was consistent with the results reported by Tefekli and colleagues^9^ and Nalbant and colleagues,^10^ but different from those reported by Akman and colleagues.^8^ Possible reasons may be that some MetS components may have been well controlled preoperatively in the study by Akman and colleagues.^8^

The limitation of our study is the lack of stone composition. Staghorn stones mainly consist of calcium oxalate (68%), uric acid (28%), and struvite or calcium phosphate (24%),^28^ which is similar to the results of a recent Chinese systemic review of 11,157 general renal stones with calcium oxalate (51.6%–67.9%), uric acid (5.7%–19.3%), and infectious stone (18.3%–24.8%).^29^ Additionally, patient inclusion criteria were limited to renal staghorn stones. Therefore, the complication rates in our study were higher than those of PCNL on smaller and simpler stones. Notably, the diagnostic criteria of MetS does not reach the criteria for HT (≥140/90 mm Hg *vs* >130/85 mm Hg) and DM (FPG ≥7.0 mmol/L *vs* >5.6 mmol/L), meaning that MetS is not a set of definite diseases, but disorders or weak performance status started from subhealth. Our results recommended not to underestimate subhealth disorders. For simple and short operations, MetS may not significantly affect the operations, but for large surgeries for complex diseases, MetS should be pointed out as a considerable potential risk.

## Conclusion

The number of MetS components should be regarded as the central role in assessing both inner severity of MetS and outer risk for PCNL. The number of MetS components is recommended to be calculated on a scale of 0 to 5. Three, four, and five MetS components increased risk for PCNL in a stepwise manner regardless of the presence or absence of obesity. Uncontrolled MetS components should be considered as a relative contraindication of PCNL and should be controlled preoperatively.

## Abbreviations Used

BMI: body mass index
BP: blood pressure
CI: confidence interval
CT: computed tomography
DM: diabetes mellitus
eGFR: estimated glomerular filtration rate
FPG: fasting plasma glucose
HDL: high-density lipoprotein cholesterol
HT: hypertension
MetS: metabolic syndrome
MI: myocardial infarction
OR: odds ratio
PCNL: percutaneous nephrolithotomy
SD: standard deviation
SFR: stone-free rate
TG: triglycerides
